# Characteristics, Composition and Oxidative Stability of *Lannea microcarpa* Seed and Seed Oil

**DOI:** 10.3390/molecules19022684

**Published:** 2014-02-24

**Authors:** Patrice Bazongo, Imaël Henri Nestor Bassolé, Søren Nielsen, Adama Hilou, Mamoudou Hama Dicko, Vijai K. S. Shukla

**Affiliations:** 1 Laboratoire de Biochimie Alimentaire, Enzymologie, Biotechnologie Industrielle et Bioinformatique (Laboratoire BAEBIB), Department of Biochemistry and Microbiology, Université de Ouagadougou, Ouagadougou 03 03 BP 7021, Burkina Faso; E-Mails: pathcobaz@yahoo.fr (P.B.); mdicko@univ-ouaga.bf (M.H.D.); 2 International Food Science Centre (IFSC A/S), Sønderskovvej, Lystrup 7 DK-8520, Denmark; E-Mails: sn@ifsc.dk (S.N.); gamma1948@hotmail.com (V.K.S.S.); 3 Laboratoire de Biochimie et Chimie Appliquées (LABIOCA), UFR/SVT, Université de Ouagadougou, Ouagadougou 09 09 BP 848, Burkina Faso; E-Mail: hiloudio@gmail.com

**Keywords:** *Lannea microcarpa*, seed oil, oxidative stability, fatty acids, triacylglycerols, phenolic compounds

## Abstract

The proximate composition of seeds and main physicochemical properties and thermal stability of oil extracted from *Lannea microcarpa* seeds were evaluated. The percentage composition of the seeds was: ash (3.11%), crude oil (64.90%), protein (21.14%), total carbohydrate (10.85%) and moisture (3.24%). Physicochemical properties of the oil were: refractive index, 1.473; melting point, 22.60°C; saponification value, 194.23 mg of KOH/g of oil; iodine value, 61.33 g of I_2_/100 g of oil; acid value, 1.21 mg of KOH/g of oil; peroxide value, 1.48 meq of O_2_/kg of oil and oxidative stability index, 43.20 h. Oleic (43.45%), palmitic (34.45%), linoleic (11.20%) and stearic (8.35%) acids were the most dominant fatty acids. Triacylglycerols with equivalent carbon number (ECN) 48 and ECN 46 were dominant (46.96% and 37.31%, respectively). The major triacylglycerol constituents were palmitoyl diolein (POO) (21.23%), followed by dipalmitoyl olein (POP) (16.47%), palmitoyl linoleyl olein (PLO) (12.03%), dipalmitoyl linolein (PLP) (10.85%) and dioleoyl linolein (LOO) (9.30%). The total polyphenol and tocopherol contents were 1.39 mg GAE g^−1^ DW and 578.56 ppm, respectively. γ-Tocopherol was the major tocopherol (437.23 ppm). These analytical results indicated that the *L. microcarpa* seed oil could be used as a frying oil and in the cosmetic industry.

## 1. Introduction

Recent studies have shown that some seeds might have useful applications in animal feeds, based on their nutritional values, and as raw materials for paint formulations, based on the amount and nature of their constituent oils [1]. Thus, scientists of various specialties have invested effort to highlight these new products and their industrial applications, such as for cooking, pharmaceutical, or cosmetic purposes.

Anarcadiaceae family species are well represented in Africa and have sizes between range between those of trees and small shrubs. The genus *Lannea* belongs to the Anacardiaceae family and comprises about 40 species which are restricted to Africa [2].

*Lannea microcarpa*, commonly known as African grape*,* is widely distributed in the sub-Saharan region from Senegal to Cameroon. Traditional remedies prepared from its leaves, bark, roots and fruits are used to treat mouth blisters, rheumatism, sore throats, dysentery, conjunctivitis, stomatitis, skin eruptions, and ulcers [2,3]. The fruit is eaten raw or dried, and a number of fermented or soft drinks are produced from its juice. Important concentrations of phenolic compounds with strong antioxidant capacities have been found in the fruit (1005.75 mg/100 g of fruit) [4]. 4'-methoxymyricetin 3-*O*-*α*-L-rhamnopyranoside, myricetin 3-*O*-*α*-L-rhamnopyranoside, myricetin 3-*O*-*β*-D-glucopyranoside, vitexin, isovitexin, gallic acid, and epicatechin have been identified as major constituents of the leaf extracts [3]. Partial reports are available on its seed and seed oil protein and lipid contents and their compositions [5], however, literature reports concerning the detailed lipid, triacylglycerol and phenolic compounds composition and oil stability are rather lacking. This study was therefore conducted to document the characteristics, composition and oxidative stability of *L. microcarpa* seeds and seed oil in order to determine their potential to be commercially exploited for industrial applications.

## 2. Results and Discussion

### 2.1. Proximate Composition of Seeds

The proximate composition of *L. microcarpa seeds* is shown in Table 1. The seeds contained 3.24% of moisture, 3.11% of ash, 10.85% of total carbohydrates, 21.14% of crude protein and 64.90% of crude oil. The crude oil and protein contents were lower and higher respectively than those previously reported by Glew *et al.* [5]. The seeds can be considered as a good source of oil and protein. The moisture content was beneficial for prolonging the shelf life of the seeds.

**Table 1:** Proximate composition of *Lannea microcarpa* seeds (g/100 g dry weight basis).

Components	Contents
Moisture (%)	3.24 ± 0.56
Proteins	21.14 ± 0.25
Lipids	64.90 ± 1.27
Carbohydrates	10.85 ± 1.56
Ash	3.11 ± 0.22

Mean ± SD, *n* = 3.

### 2.2. The Physicochemical Properties of Seed Oil

Physicochemical properties of *L. microcarpa* seed oil are listed in Table 2. The oil exhibited refractive index of 1.473 which was similar to that of *Acacia senegal* seed oil [6]. The seed oil melting point was 22.60 °C which makes it a liquid at ambient temperature. The saponification value, 194.23 mg KOH/g of oil, was lower than that reported for lauric oil (240–265 KOH/g of oil) and in the range of those of cotton seed oil (193–195) and linseed oil (193–195) [6,7]. Triacyglycerols containing short-chain fatty acids have higher saponification values than those with longer chain fatty acids [8]. The *L. microcarpa* seed oil was rich in C_16_ and C_18_ acids which explained its relatively low saponification value. The low iodine value of 61.33/100 g of oil was due to its low unsaturated fatty acid contents. The oil can be classified as a non-drying oil. The acid and peroxide values were 1.21 mg of KOH/g of oil and 1.48 meq of O_2_/kg of oil, respectively. These low values suggest that the oil can be stored for a long period without deterioration [9].

**Table 2:** Physicochemical properties of *Lannea microcarpa* seed oil.

Physicochemical properties	Value
Refractive index (25 °C)	1.473 ± 001
Melting point (°C)	22.60 ± 0.75
Saponification value (mg of KOH/g of oil)	194.23 ± 0.80
Iodine value (g of I_2_/100 g of oil)	61.33 ± 0.25
Acid value (mg of KOH/g of oil)	1.21 ± 0.01
Peroxide value (meq of O_2_/kg of oil)	1.48 ± 0.11
Oxidative stability index (h)	43.20 ± 2.00

Mean ± SD, *n* = 3.

The stability of *L. microcarpa* seed oil at 130 °C expressed as the oxidation induction time was 43.20 h. This value was higher than those of 6.10 h and 1.10 h determined at 120 °C for olive and linseed oils, respectively [10]. The high oxidation induction time confirms high oxidative stability of *L. microcarpa* seed oil. Due to its high oxidative stability of the *L. microcarpa* seed oil can be used as edible cooking or frying oil.

### 2.3. Fatty Acid Composition

The fatty acid composition of crude oil of *L. microcarpa* seeds is presented in Table 3. A total of 10 fatty acids were identified. The most abundant fatty acids were oleic (44.05%), palmitic (34.45%), linoleic (11.20%) and stearic (8.35%) acids which accounted for 98.05% of the total fatty acids. The saturated, monounsaturated and polyunsaturated fatty acids were 44.10%, 44.35% and 11.55% of the total fatty acids, respectively. The fatty acid composition of *L. microcarpa* seed oil was comparable to that of *Adansonia digitata* seed oil [11] and like that one, the *L. microcarpa* seed oil can be regarded as a oleic-palmitic oil while that reported by Glew *et al.* [5] was a palmitic-linoleic-oleic type oil. The variations in fatty acid composition, lipid and protein contents may be attributed to the seed maturity, climatic conditions, growth location, and interactions between these factors. Oleic and palmitic acids have been reported to be effective percutaneous absorption enhancers [12,13]. In addition, *microcarpa* seed oil had relative high content in linoleic acid which is the most frequently used fatty acid in cosmetic products as it moisturises the skin, aids in the healing process of dermatoses and sunburns [14]. In recent years, baobab oil has been added to the list of fixed oils commonly included in cosmetic products due to its high contents in palmitic and oleic acids [11]. Like baobab oil, the *L.* *microcarpa* seed oil thus has a good cosmetic potential.

**Table 3:** Fatty acid composition of *Lannea microcarpa* seed oil.

Fatty acid	Values
Palmitic acid 16:0	34.45 ± 0.35
Heptadecanoic acid 17:0	0.20 ± 0.00
Stearic acid 18:0	8.35 ± 0.21
Oleic acid 18:1 n – 9	43.45 ± 0.21
Oleic acid 18:1 Trans	0.6 ± 0.05
Linoleic acid 18:2 n – 6	11.20 ± 0.13
Linolenic 18:3 n – 3	0.35 ± 0.07
Eicosanoic acid 20:0	0.9 ± 0.03
Eicosenoic acid 20:1 n – 9	0.3 ± 0.01
Behenic acid 22:0	0.2 ± 0.01
SFA	44.10
MUFA	44.35
PUFA	11.55

SFA, saturated fatty acid; MUFA: monounsaturated fatty acid, PUFA: polyunsaturated fatty acid, (mean ± SD, *n* = 2).

### 2.4. Triacylglycerol (TAG) Profile

Table 4 shows the triacylglycerol composition of *L.* *microcarpa* seed oil. According to the results, the oil contained five triacylglycerol types [from equivalent carbon number (ECN) 44 to ECN 52]. Triacylglycerols with ECN 48 were dominant (46. 96%), followed by TAGs ECN 46 (37.31%), TAGs ECN 50 (10.21%), TAGs ECN 44 (4.01%) and TAGs ECN 52 (1.51%). Major TAG constituents were palmitoyl diolein (POO) (21.23%), followed by dipalmitoyl olein (POP) (16.47%), palmitoyl linoleyl olein (PLO) (12.03%), dipalmitoyl linolein (PLP) (10.85%) and dioleoyl linolein (LOO) (9.30%). The saturated fatty acids were absent from the sn-2 position and the major TAG molecular species had oleic and linoleic acids at the sn-2 position. This result reflects the high unsaturation of the oil. Good agreement between the fatty acid and TAG compositions was also found.

**Table 4:** Triglyceride composition (Mole %) of *Lannea microcarpa* seed oil.

Triglyceride	ECN	Values
POP	48	16.47 ± 0.09
POS	50	6.51 ± 0.01
SOS	52	1.51 ± 0.02
PLP	46	10.85 ± 0.07
POO	48	21.23 ± 0.53
SOO	50	3.7 ± 0.14
LLO	44	2.50 ± 0.03
PLL	46	5.13 ± 0.32
PLnO	44	1.51 ± 0.02
LOO	46	9.30 ± 0.00
PLO	46	12.03 ± 0.25
OOO	48	6.24 ± 0.34
SLO	48	3.02 ± 0.03

POP: dipalmitoyl olein; POS: palmitoyl oleoyl stearin; SOS: distearoyl olein; PLP: dipalmitoyl linolein; POO: palmitoyl diolein; SOO: stearoyl diolein; LLO: dilinoleyol olein; PLL: palmitoyl dilinolein; PLnO: palmitoyl linolenoyl olein; LOO: linoleyol diolein; PLO: palmitoyl linoleyl olein, OOO: triolein; SLO: stearoyl linoleyl olein (mean ± SD, *n* = 2).

### 2.5. Phenolic Compound Contents

The total polyphenol content of *L. microcarpa* seed oil was 1.39 mg gallic acid equivalent (GAE) g^−1^ dry weight (DW). This value is higher than those of 0.067–1.295 mg/g of oil reported for olive oil [15]. The tocopherol composition of *L.* *microcarpa* seed oil is given in Table 5. The most frequent form of tocopherol was γ-tocopherol (437.23 ppm), followed by α-tocopherol (89.40 ppm) and δ-tocopherol (51.93 ppm). β-Tocopherol was not detected. Total amount of tocopherol (578.56 ppm) in *L.* *microcarpa* seed oil was higher than that recorded in *Sclerocarya birrea* seed oil (137 ppm) and olive oil (100–300 ppm) [16,17].

**Table 5:** Total polyphenol content and tocopherol Composition of *Lannea microcarpa* seed oil.

Components	Values
Total polyphenol (mg GAE g^−1^ DW)	1.39 ± 0.05
α-tocopherol (ppm)	89.40 ± 0.57
β-tocopherol (ppm)	0.00 ± 0.00
γ-tocopherol (ppm)	437.23 ±1.73
δ-tocopherol (ppm)	51.93 ± 0.11
Total tocopherol (ppm)	578.56 ± 2.19

Mean ± SD, *n* = 2.

The high oxidative stability of *L.* *microcarpa* can be due to its polyphenol and tocopherol contents. Gutierrez *et al.* [18] observed that removal of polyphenol from olive without altering other antioxidant components lead to the loss of 50% of the oil stability. Huang *et al.* [19] have reported the contribution of tocopherol to corn oil stability. The antioxidant activity of tocopherol in oils is greatly dependent on its composition. The same author noted that at 100 ppm, α-tocopherol exerted the best antioxidant activity in corn oil when compared with γ-tocopherol and δ-tocopherol. Baldioli *et al.* [20] reported the synergistic effect of α-tocopherol with hydrophilic phenols on the oxidative stability in virgin olive oils.

## 3. Experimental Section

### 3.1. Plant Material

Fruits of *L. microcarpa* (1.5 kg) at the same maturity stage were collected on ground at Djanga (latitude 10.37 N; longitude 4.47 W) and Bérégadougou (latitude 10.49 N; longitude 4.47 W) villages in the Southwest of Burkina Faso. Before their transport to the laboratory, the fruits were hand sorted to eliminate damaged ones. Prior to any analysis, fruit samples were washed with glass-distilled water, drained, and air dried under laboratory conditions (22–23 °C) for one week. The dried seeds were milled with a Moulinex grinder (GT550, Zurich, Switzerland) then sieved using a 1 mm mesh sieve and stored at −18 °C until analyses.

### 3.2. Seed Analysis

Moisture, protein, ash and lipid contents were determined according to the protocols established by the Association of Official Analytical Chemists [21]. Moisture was determined gravimetrically after drying the sample overnight at 105 °C. Total protein content (% total nitrogen × 6.25) was established by the Kjeldahl method. Ash was quantified after incinerating the sample overnight at 550 °C. Total lipids were determined by Soxhlet extraction with petroleum ether for 6 h, after which the solvent was removed using a rotary vacuum evaporator. Carbohydrate content (on dry weight basis) was estimated by difference of mean values: 100 − (sum of percentages of ash, protein and lipids) [22].

### 3.3. Physicochemical Analysis of the Oil

Official methods of the American Oil Chemists’ Society were used for the determination of saponification (Cd 3–25), iodine (Cd 1–25), peroxide (Cd 8–53) and acid (Ca 3a–63) values [23]. Refractive index was measured at 25 °C with an Abbe refractometer (Atago 2T, Tokyo, Japan). The oxidative stability index (OSI) was evaluated using Rancimat method. Stability was measured with a 743 rancimat instrument (Metrohm, Herisau, Switzerland) using an oil sample of 3 g, warmed to 130 °C and an air flow rate of 20 L/h. Stability was expressed as induction time (h). All the analyses were carried out in triplicate and the results are expressed as the mean ± standard deviation (x ± SD).

### 3.4. Fatty Acid Analysis

The oil was transesterified with boron trifluoride and gas liquid chromatography (GLC) of fatty acid methyl esters were performed using a Perkin Elmer 8500 system fitted with a programmable temperature vaporizer (PTV) injector and a flame ionization detector (FID) with a data processor [24]. Helium was used as carrier gas. The column temperature was initially maintained at 175 °C and then raised by 1 °C/min to 200 °C held for 10 min and then raised by 2 °C/min to 230 °C and held for 20 min. The PTV injector was initially maintained at 45 °C and immediately after the injection raised to 250 °C. The FID was kept at 300 °C. The capillary column (30.0 m × 0.25 mm) employed was CP Sil 88 (Chrompack/Varian Instruments, Walnut Creek, CA, USA) with a film thickness at 0.2 µm. The peaks were identified by comparing retention times with authentic fatty acid methyl esters. This experiment was carried out in duplicate.

### 3.5. Triglyceride Composition Analysis

Triglyceride compositions were determined according to method describe by Shukla *et al.* [25]. The HPLC system consisted of a Spectra Physics (SP 8000) chromatograph (Spectra-Physics, Santa Clara, CA, USA), coupled with a Pye Unicam LC-UV detector (Pye Unicam Ltd., Cambridge, UK) at 220 nm, and a Rheodyne loop (5 µL) injector (model 7125, Rheodyne, Cotati, CA, USA). A model 9800 differential refractometer (Knauer, Berlin, Germany)was used for refractive index measurements. An SP 8000 electronic integrator was used to obtain accurate retention times at a chart speed of 0.25 cm/min. The columns used for the separations consisted of two 150 mm × 4.5 mm I.D. Spherisorb S3 ODS 2 (Phase Separations, Clwyd, UK) arranged in series and packed with 3 µm C_18_ bonded phase particles. The columns were maintained at 20 °C by coupling the column oven with a Hetofrig cooling system (type 03 PF 623 CB 11, Birkerod, Denmark). The mobile phase consisted of acetonitrile-tetrahydrofuran (70:30 v/v), both of HPLC grade (Rathburn, Walkerburn, U.K.), and the flow rate was 1.0 mL/min. The sample size was 5–10 µL of ca. 10% solutions of triglycerides. Triglyceride isomers were identified by comparison of their retention time to those of cocoa butter obtained under similar analytical conditions [24]. The analysis was done in duplicate.

### 3.6. Determination of Total Polyphenol Content (TPC)

TPC in the oil was evaluated using the Folin–Ciocalteu assay, as adapted by Gharibzahedi *et al.* [26], with some modifications. Distilled water (3.16 mL) was mixed with a DMSO solution of the oil (40 µL) and then Folin Ciocalteu reagent (200 µL) was added to the solution. This mixture was allowed to stand at room temperature for 5 min and then 600 µLof 20% sodium carbonate solution was added. After 2 h of incubation, the absorbances were measured at 765 nm using a UV–visible spectrophotometer (Epoch, Biotek, Winooski, VT, USA). Results were expressed as mg GAE per g of oil. This experiment was performed in duplicate.

### 3.7. Tocopherol Analysis

Tocopherols were separated and quantified by HPLC, according to AOCS method Ce 8–89 [23]. Oil was dissolved in *n*-hexane and submitted directly to HPLC analysis. A Perkin Elmer Fluorescence detector Series 200 and a Rheodyne 7125 Injector (IDEX Health and Science, Oak Harbor, WA, USA) equipped with a 20 µL loop were used. Excitation wavelength was 290 nm and Emission wavelength was 330 nm. The solvent system used was water saturated hexane, hexane and propan-2-ol (49.55:49.55:0.9). The columns consisted of 2 × 150 mm 4.6 mm I.D. packed with 3 µm CN particles [27]. A mixed tocopherols standard was used, which contained α-, β-, γ-, δ-tocopherols (Sigma-Aldrich, Inc., St. Louis, MO, USA). The tocopherol contents of samples were quantified by the external standard method. The analysis was done in duplicate.

## 4. Conclusions

In this study, the composition and physicochemical characteristics of *L. microcarpa* seed and seed oil from Burkina Faso were evaluated. Seeds were characterized by high crude oil and good protein contents that make them valuable as animal feed or for human nutrition. High oxidative stability of the oil due to its polyphenol and tocopherol contents was comparable to those of *Moringa pterygosperma*, *Sclerocarya birrea* seed and *Olea europaea* fruit oils. The obtained data suggest that the *L. microcarpa* seed oil has a good potential as a frying oil and can be used in the cosmetic industry.
